# Epilepsy is associated with the accelerated aging of brain activity in sleep

**DOI:** 10.3389/fphys.2024.1458592

**Published:** 2024-11-28

**Authors:** Peter N. Hadar, Mike Westmeijer, Haoqi Sun, Erik-Jan Meulenbrugge, Jin Jing, Luis Paixao, Ryan A. Tesh, Madalena Da Silva Cardoso, Pierrick Arnal, Rhoda Au, Chol Shin, Soriul Kim, Robert J. Thomas, Sydney S. Cash, M. Brandon Westover

**Affiliations:** ^1^ Department of Neurology, Massachusetts General Hospital (MGH), Boston, MA, United States; ^2^ Utrecht University, Utrecht, Netherlands; ^3^ Department of Neurology, Beth Israel Deaconess Medical Center, Boston, MA, United States; ^4^ Department of Radiology, NYU-Langone Medical Center, New York, NY, United States; ^5^ Dreem, Paris, France; ^6^ Department of Epidemiology, Boston University School of Medicine, Boston, MA, United States; ^7^ Institute of Human Genomic Study, College of Medicine, Korea University, Seoul, Republic of Korea; ^8^ Biomedical Research Center, Korea University Ansan Hospital, Ansan, Republic of Korea; ^9^ Department of Medicine, Division of Pulmonary, Critical Care & Sleep, Beth Israel Deaconess Medical Center, Boston, MA, United States

**Keywords:** Epilepsy, brain age, sleep, cognition, EEG

## Abstract

**Objective:**

Although seizures are the cardinal feature, epilepsy is associated with other forms of brain dysfunction including impaired cognition, abnormal sleep, and increased risk of developing dementia. We hypothesized that, given the widespread neurologic dysfunction caused by epilepsy, accelerated brain aging would be seen. We measured the sleep-based brain age index (BAI) in a diverse group of patients with epilepsy. The BAI is a machine learning-based biomarker that measures how much the brain activity of a person during overnight sleep deviates from chronological age-based norms.

**Methods:**

This case–control study drew information of age-matched controls without epilepsy from home sleep monitoring volunteers and from non-epilepsy patients with Sleep Lab testing. Patients with epilepsy underwent in-patient monitoring and were classified by epilepsy type and seizure burden. The primary outcomes measured were BAI, processed from electroencephalograms, and epilepsy severity metrics (years with epilepsy, seizure frequency standardized by year, and seizure burden [number of seizures in life]). Subanalyses were conducted on a subset with NIH Toolbox cognitive testing for total, fluid, and crystallized composite cognition.

**Results:**

138 patients with epilepsy (32 exclusively focal and 106 generalizable [focal seizures with secondary generalization]) underwent in-patient monitoring, and age-matched, non-epilepsy controls were analyzed. The mean BAI was higher in epilepsy patients vs controls and differed by epilepsy type: −0.05 years (controls) versus 5.02 years (all epilepsy, *p* < 0.001), 5.53 years (generalizable, *p* < 0.001), and 3.34 years (focal, *p* = 0.03). Sleep architecture was disrupted in epilepsy, especially in generalizable epilepsy. A higher BAI was positively associated with increased lifetime seizure burden in focal and generalizable epilepsies and associated with lower crystallized cognition. Lifetime seizure burden was inversely correlated with fluid, crystallized, and composite cognition.

**Significance:**

Epilepsy is associated with accelerated brain aging. Higher brain age indices are associated with poorer cognition and more severe epilepsy, specifically generalizability and higher seizure burden. These findings strengthen the use of the sleep-derived, electroencephalography-based BAI as a biomarker for cognitive dysfunction in epilepsy.

## Key points

1. The brain age index (BAI) is a machine learning-based sleep EEG biomarker associated with cognitive dysfunction and dementia.
2. This study demonstrates that epilepsy is associated with accelerated brain aging, based on higher brain age indices.
3. High brain age indices, indicating accelerated brain aging, are associated with poorer cognition in patients with epilepsy.
4. High brain age indices are associated with more severe epilepsy, both in terms of generalizability and increased seizure burden.
5. Future implementations of the BAI could promote early interventions to slow cognitive dysfunction in patients with epilepsy.

## Introduction

Epilepsy affects more than 45 million people worldwide (Beghi et al., 2019; Murray et al., 2012). Although seizures are the cardinal feature, brain dysfunction in epilepsy takes several additional forms. People with epilepsy experience increased prevalence of psychiatric disorders, like depression and anxiety, and cognitive impairment, including problems with concentration, processing speed, and memory (Beghi, 2016; Keezer et al., 2016; Kanner, 2016; Rauh et al., 2022; Kanner, 2003). Brain dysfunction is accompanied by abnormalities in the interictal brain activity, including epileptiform discharges (spikes and sharp waves), and abnormalities during sleep such as reduced rapid eye movement (REM) and deep (stage N3) sleep and increased sleep fragmentation (Louis, 2011; Gibbon et al., 2019; Rossi et al., 2020; Halasz and Szucs, 2017; Malow et al., 1999; Fountain et al., 1998; Nascimento and Nath, 2020; Seneviratne et al., 2016). People with epilepsy also have increased lifetime risk of developing dementia (Degiorgio et al., 2020; Johnson et al., 2020). Recent work from our laboratory has demonstrated that early detection of decline in cognitive function is possible using macro- and micro-structural sleep features to distinguish between subjects with mild cognitive impairment and subjects with normal cognitive ability (Ye et al., 2023). Early detection of cognitive dysfunction in epilepsy could potentially promote additional testing and interventions to try to slow the decline early and improve the quality of life, especially through cognitive prehabilitation and possibly with medications in the future, as seen recently in mild cognitive impairment with lecanemab (Farina et al., 2015; Baxendale, 2020; Cummings et al., 2023).

The concept that a person’s “brain age” can be greater than a person’s chronological age has been proposed to account for brain dysfunction in a variety of chronic medical conditions (Cole et al., 2019). The sleep EEG-based BAI is a machine learning model that quantifies how much an individual’s calculated brain age (BA) deviates from chronological age (Sun et al., 2019). Our prior work has demonstrated that excess BAI is associated with psychiatric and neurological diseases, diabetes, hypertension, early and late-stage dementia (Ye et al., 2020), HIV infection (Leone et al., 2021), and reduced life expectancy (Sun et al., 2019; Paixao et al., 2020; Petit et al., 2004).

Here, we test the hypothesis that cognitive impairment in epilepsy is associated with accelerated sleep-based brain aging (increased BAI). We also investigate whether increased BAI in epilepsy correlates with epilepsy type, frequency of seizures, and lifetime seizure burden (National Institutes of Health; Weintraub et al., 2014).

## Methods

### Study design and ethics approval

This cross-sectional retrospective study was conducted on data from patients at Massachusetts General Hospital (MGH). Prospective assessment of cognitive performance using the NIH Toolbox was performed initially until enrollment had to be stopped because of the COVID-19 pandemic; for these cases, verbal consent was obtained under a protocol approved by the Institutional Review Board (IRB) and listed as MGB IRB 2013P001024 and 2017P002444. We further included a retrospective cohort of patients; the IRB waived the requirement for informed consent for this component of the study.

### Patients with epilepsy

Inclusion criteria for epilepsy patients were as follows: adults (≥18 years old) with diagnosed epilepsy who underwent continuous EEG monitoring in the epilepsy monitoring unit (EMU) and had a detailed evaluation for epilepsy, including an MRI brain scan and neuropsychological testing within 6 months of their stay in the EMU. Exclusion criteria are detailed in Supplementary Figure S1. EEG recordings were obtained from the Massachusetts General Brigham EEG database. Clinical data (i.e., demographics, medication, seizure semiology, and medical history) were extracted from EMU admission and clinical neurology notes. Prospective enrollment for patients admitted to the EMU, who were then administered the NIH toolbox, took place between 14 February 2019 and 17 March 2020. Retrospective enrollment included the period from 1 January 2016 to 21 July 2022 and did not involve administration of the NIH toolbox.

### Matched controls

The matched controls were from two groups. The first group of non-epilepsy subjects originated from the Dreem cohort, including a set of sleep-EEG recordings from volunteers who wore a portable home EEG device for 7 days on average (n = 1,077). The other group of non-epilepsy subjects were extracted from the Sleep Lab cohort with the NIH Toolbox Cognitive Battery test results available (n = 112). Non-epilepsy subjects from both groups had to pass a set of exclusion criteria to be considered for matching (Supplementary Figure S1). Individuals were matched based on age and sex (Sen et al., 2020; Cole et al., 2017; Eggert et al., 2021; Hu et al., 2021). We used weighted full matching to match the group of patients with epilepsy with two groups of non-epilepsy subjects who also underwent long-term EEG monitoring; this method minimizes propensity score differences between cases and controls while maintaining the highest number of control individuals (Stuart, 2010). Note that when we analyze cognition, only the Sleep Lab cohort subjects are used as matched controls.

### EEG preprocessing and artifact removal

EEG electrodes were placed on the scalp as part of clinical care following the international 10–20 system. EEG recordings were started on admission to the EMU and continued throughout hospitalization. For analysis, EEG signals were down-sampled from 512 Hz to 200 Hz and notch-filtered at 60 Hz to remove line noise. We used a previously published neural network to perform automated sleep scoring; this method segments the EEG into non-overlapping 30-s epochs and classifies each epoch as one of five sleep stages: awake (A), rapid eye movement (REM) sleep, non-rapid eye movement (NREM) stage 1 (N1), NREM stage 2 (N2), or NREM stage 3 (N3) (Jaoude et al., 2020). Artifact removal was accomplished with two complementary methods: (1) epochs with maximum absolute amplitude >500 μV or standard deviation <1 μV were removed. (2) We trained a linear discriminant analysis (LDA) classifier to classify each epoch into artifact vs. clean (no artifact) based on the total power and the second-order difference (for abrupt non-physiological changes) of the spectrum. (3) Epochs scored as awake with eyes open, characterized by blinking patterns and a reactive posterior dominant rhythm, were removed. (4) SpikeNET, a previously published machine learning algorithm, was used to exclude epochs with interictal epileptiform discharges (IEDs) (Jing et al., 2020). (5) SPARCnet, a recently developed deep neural network, was used to exclude epochs with seizures and seizure-like events (Jing et al., 2023). These were further reviewed manually by an epileptologist (PNH, one of the authors, below). Generalized rhythmic delta activity (GRDA) was not counted as a seizure-like pattern due to its similarity to N3 sleep.

### Calculation of the BAI

BA was calculated using a machine learning model developed by Sun et al., which uses the sleep-EEG data from six scalp electrodes as the input: two frontal electrodes (F3 and F4), two central electrodes (C3 and C4), and two occipital electrodes (O1 and O2) (Sun et al., 2019). The signals were bandpass-filtered between 0.5 and 20 Hz, as was performed in the BAI model. A total of 510 sleep microstructure features concatenated from the five sleep stages were used to calculate the BA, including spectral band powers and their ratios and signal complexity measures, and were log-transformed to approximate a Gaussian distribution and z-transformed to have zero mean and unit standard deviation, as per prior BA publications from our laboratory (Sun et al., 2019). To reduce night-to-night variability of the sleep-based BA, we averaged BA estimates across all nights of available EEG recordings during the EMU stay for each patient (Sun et al., 2019). BAI is computed as BA minus chronological age (CA, i.e., BAI = BA–CA). The top 15 sleep features ranked by *t* test *p*-value in ascending order were reported.

### EEG spectrograms

EEG spectrograms were computed for each night (EEGs within 11 p.m. to 7 a.m.) for two purposes: (1) to manually ensure any seizure activity was excluded from the analysis, as the BAI was designed to be used only on interictal sleep data, and (2) to visualize the representative spectrograms and hypnograms. The spectrogram consisted of spectra for each 30-s epoch. The spectra were computed using multitaper spectral estimation with 0.67 Hz frequency resolution using 19 tapers. To select representative spectrograms and hypnograms, sleep EEG features used by the BAI model were standardized, and mean values were calculated for the epilepsy patients and sleep lab controls. For each group, we selected the 10 participants with spectral features closest (i.e., based on Euclidean distance) to the group mean. Next, we manually selected three spectrograms and hypnograms for epilepsy patients who were most visually representative for the low, average, and high BAI groups (Supplementary Figure S2).

### Cognitive tests

Cognitive performance was measured for a subset of epilepsy patients (n = 39) and sleep lab controls (n = 112, below) using the NIH Toolbox cognitive battery. There was no cognitive score available for the Dreem controls. The NIH Toolbox is a validated and normed assessment of behavioral and neurological functions and offers reliable tools for assessing cognition (National Institutes of Health; Weintraub et al., 2014). The cognitive battery consists of five subtests that measure fluid cognition and two subtests that measure crystallized cognitive abilities. Total fluid cognition and total crystallized scores were calculated based on the average standard scores of the subtests. Total composite cognition is a weighted average of total fluid cognition and total crystallized cognition.

### Epilepsy metrics

Three quantitative measures of epilepsy severity were determined to capture acute and chronic phases of the disease: seizure frequency, years of epilepsy, and lifetime seizure burden. Pertinent patient information was collected from a combination of initial EMU admission note, an epilepsy surgical conference discussion note (when available), and a recent outpatient epilepsy note in the 3 months prior to EMU admission. All patients with epilepsy were first analyzed as a group and then divided into two mutually exclusive subgroups based on seizure semiology: (1) generalizable seizures (any patient who had focal seizures with secondary generalization) or (2) exclusively focal seizures. This designation was determined based on the chart review of outpatient notes and of EEG and EMU reports.

The seizure frequency per year was tabulated by the type of seizure. However, as this metric only provided a recent snapshot of seizure burden, chronic metrics of seizure burden were created to help quantify long-term effects of epilepsy. Years of epilepsy were determined by subtracting the age at the time of EMU admission from the age at which the first seizure occurred. Notably, this did not include the frequency of seizures, which differed significantly between patients. Lifetime seizure burden, a metric meant to more closely approximate the chronic effects of epilepsy, was defined as the number of seizures a patient suffered throughout life to date. This measure was approximated by the documented seizure frequency (based on the chart review of notes within 6 months of EMU admission) if available or by multiplying the years of epilepsy by the previously calculated yearly seizure frequency.

### Statistical analyses

Baseline patient characteristics were analyzed using the chi-square (categorical variables) or Mann–Whitney (continuous variables) tests, comparing demographic and medical history measures between those with focal and generalizable epilepsy.

A *t* test was used to investigate the difference in the sleep stage distribution between patient groups. First, comparisons were made between all patients with epilepsy and the sleep lab controls, and next between epilepsy types (i.e., generalizable epilepsy and focal). Next, the difference in mean BAI (which is already log-transformed and z-transformed) between patients with epilepsy and the matched controls was investigated using a *t* test. The pairwise comparison of the mean BAI between the different epilepsy subgroups and healthy controls was conducted, followed by correlating the lifetime seizure burden to BAI with a Pearson’s correlation coefficient *r.* Next, the correlation between lifetime seizure burden and cognitive performance, as well as the correlation between BAI and cognitive performance, was examined using Pearson’s *r.* Finally, the strength of the correlation between BAI and cognitive performance was compared between patients and controls using analysis of covariance (ANCOVA) using cognitive performance as the outcome variable and BAI and case/control status as covariates.

Whenever cognitive impairment was assessed, the 39-subject epilepsy cohort that underwent prospective NIH toolbox testing was used; in other cases when cognitive impairment was not assessed, the entire 138-subject epilepsy cohort was used.

Statistical significance was defined as *p*-value <0.05. The BAI is presented as mean ± standard error (SE). Statistical analyses were performed using RStudio version 4.2.1 and Python version 3.7 (Python Software Foundation).

### Data availability

The de-identified data and code to reproduce the results will be available after the time of publication. Data will be found at the Brain Data Science Platform (https://bdsp.io/), and code will be found at https://github.com/bdsp-core/BAI-EPILEPSY.

## Results

### Baseline characteristics

We prospectively enrolled 40 patients to perform neuropsychological testing with the NIH toolbox. We further retrospectively identified 100 patients from the epilepsy monitoring unit who met inclusion criteria for BA analysis; these patients did not have NIH toolbox data available. Two patients were excluded as they were not diagnosed with epilepsy, leaving 39 with neuropsychological testing and 99 without neuropsychological testing. The final cohort consisted of 138 participants (54.3% women) with an average age of 39.6 (standard deviation 13.4 years). The baseline patient characteristics are shown in Table 1. Control subjects from the Dreem and Sleep Lab non-epilepsy groups were selected from 2,316 to 8,673 subjects, respectively. After applying the exclusion criteria, there were 1,077 Dreem and 112 Sleep Lab patients remaining who served as controls (Supplementary Figure S1).

**TABLE 1:** Baseline characteristics epilepsy patients (N = 138).

	Focal (n = 32)	Generalizable (n = 106)	*p*-value^a^
Age, years	42.9 ± 15.1	38.6 ± 12.8	0.2
BMI	28.9 ± 7.0	27.6 ± 7.9	0.2
Sex, male	9 (28.1)	54 (50.9)	**0.023**
Years of education	14.7 ± 3.2	13.6 ± 2.5	0.12
Race			0.3
White	25 (78.1)	84 (79.2)	
Hispanic/Latino	1 (3.1)	11 (10.4)	
Asian	3 (9.4)	5 (4.7)	
Black	1 (3.1)	4 (3.8)	
Other/unknown	2 (6.2)	2 (1.9)	
Nights of EEG recordings	5.6 ± 3.7	6.1 ± 3.5	0.4
Years of epilepsy	13.5 ± 12.7	14.5 ± 13.5	0.5
Number of AEDs	2.3 ± 1.0	2.3 ± 1.0	0.9
Seizure frequency per year	288.8 ± 615.4	290.2 ± 558.1	0.7
Epilepsy risk factors			
History of psychiatric diseases	17 (53.1)	59 (55.7)	0.8
Family history of epilepsy	8 (25.0)	23 (21.7)	0.7
Structural (inc. stroke, surgery, brain anomalies)	10 (31.2)	32 (30.2)	0.9
Trauma with LOC	7 (21.9)	20 (18.9)	0.7
History of substance abuse	6 (18.8)	24 (22.6)	0.6
Developmental delay	4 (12.5)	7 (6.6)	0.3
Febrile seizures	2 (6.2)	5 (4.7)	0.7
Medical history			
Brain surgery	6 (18.8)	19 (17.9)	0.9
Cardiovascular disease	8 (25.0)	12 (11.3)	0.082
Diabetes	3 (9.4)	4 (3.8)	0.4
Stroke	3 (9.4)	4 (3.8)	0.4
Current smoker	3 (9.4)	21 (19.8)	0.2
Ex-smoker	8 (25.0)	27 (25.5)	0.9
Psychiatric History	17 (53.1)	59 (55.7)	0.8
Depression	14 (43.8)	53 (50.0)	0.5
Anxiety	9 (28.1)	26 (24.5)	0.7
ADHD	2 (6.2)	11 (10.4)	0.7
PTSD	3 (9.4)	7 (6.6)	0.7
Psychotic episode	1 (3.1)	7 (6.6)	0.7
OCD	1 (3.1)	5 (4.7)	0.9
Bipolar disorder	0 (0.0)	3 (2.8)	0.9
Borderline disorder	0 (0.0)	3 (2.8)	0.9
Schizoaffective disorder	0 (0.0)	1 (0.9)	0.9
Schizophrenia	0 (0.0)	1 (0.9)	0.9
Sleep disorders	6 (18.8)	24 (22.6)	0.6
Sleep apnea	2 (6.2)	18 (17.0)	0.2
Restless legs syndrome	0 (0.0)	3 (2.8)	0.9
Insomnia	5 (15.6)	5 (4.7)	0.052
Parasomnia	0 (0.0)	1 (0.9)	0.9

All values given as mean ± standard deviation (continuous) or number (percentage, categorical).

^a^ *p*-value for Mann-Whitney (continuous) or Chi-Square/Fisher Exact (categorical) tests, significant (bolded) for p<0.05.

Abbreviations: SD, standard deviation; IQR, interquartile range; AEDs, Antiepileptic Drugs; GTC, Generalized Tonic-Clonic; CPS, Complex Partial Seizures; SPS, Simple Partial Seizures; ADHD, Attention-deficit/hyperactivity disorder; OCD, Obsessive-Compulsive Disorder; PTSD, Post-traumatic stress disorder.

### Sleep EEG macrostructural and microstructural features in epilepsy

**FIGURE 1:**
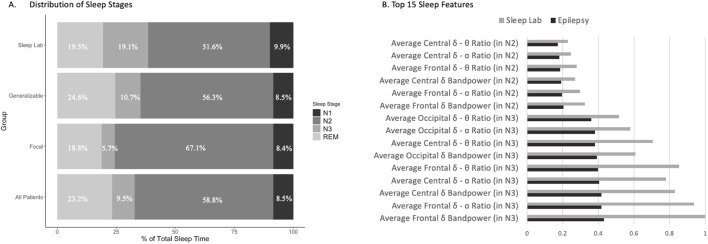
Correlation between epilepsy vs. sleep stage and EEG features. **(A)** Sleep macrostructure features, specifically sleep stages Nl, N2, N3, and REM, were identified for the Sleep Lab control group, as well as for generalizable epilepsy patients, focal epilepsy patients, and the combined all epilepsy patients. Of note, there is a statistically significant decrease in N3 sleep in all epilepsy patients relative to controls, while the differences between the remaining sleep stages are not statistically significant. **(B)** The top 15 microstructural features based on sleep EEG that differed between the patients with epilepsy and Dreem controls were noted, with the most prominent features including average central delta–theta ratio in N2 sleep, average central delta–alpha ratio in N2 sleep, and average frontal delta–alpha ratio in N2 sleep.

Correlations between epilepsy vs sleep stage distribution (sleep macrostructure) and EEG features (sleep microstructure) are shown in Figure 1. Overall, the 138 patients with epilepsy showed a reduced percentage of deep sleep (N3) (9.5% vs. 19.1% in Sleep Lab controls, *p* < 0.001); proportions for other stages were similar (8.5%, 58.8%, and 23.2% for N1, N2, and REM, respectively, vs. 9.9%, 51.6%, and 19.5% for controls, *p* > 0.05 for all).

Within the epilepsy group (Figure 1A), sleep stage proportions for the generalizable seizure group (n = 106) for N1, N2, N3, and REM sleep were 8.5%, 56.3%, 10.7%, and 24.6%, respectively; whereas for the focal seizure group (n = 32), these were 8.4%, 67.1%, 5.7%, and 18.8%, respectively. There was a statistically significant increase in the proportion of time spent in N3 and REM sleep in the generalizable seizures group vs the focal epilepsy group (*p* < 0.01) and a significant decrease in the proportion of time spent in N2 sleep in the generalizable seizure group compared to the focal group (*p* < 0.001). There was no difference seen in the proportion of time spent in N1 sleep (*p* = 0.95).

Differences in the top 15 most significantly different sleep EEG (microstructure) features between all 138 epilepsy patients and Sleep Lab healthy controls are shown in Figure 1B. These EEG features included average band powers and ratios of power in different EEG frequency bands (θ, δ, and α EEG frequencies). It is notable that the top differences involved N2 and N3 sleep features. N3 sleep features, such as frontal δ band power and frontal δ-α ratio, were highly predictive features for distinguishing sleep stages between epilepsy patients and Sleep Lab controls. Supplementary Figure S2 depicts representative hypnograms and spectrograms for epilepsy patients with low, average, and high BAI.

### Association between BAI and epilepsy

**FIGURE 2:**
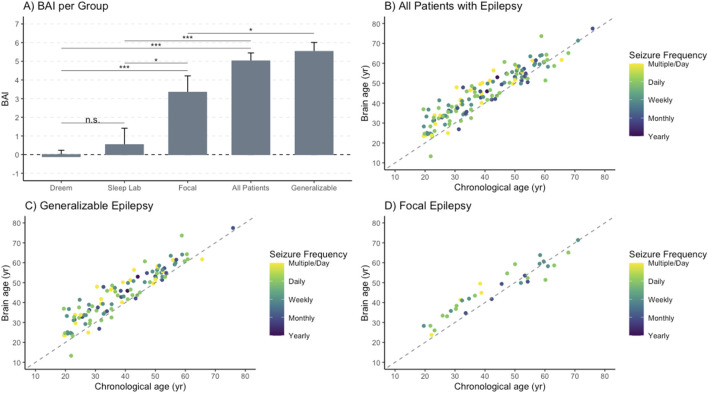
Brain age is positively associated with chronological age for patients with epilepsy. Brain age was calculated using our algorithm and compared to chronological age in patients with epilepsy, in subgroups of patients with generalizable epilepsy and focal epilepsy, and in control groups consisting of the Dreem cohort and Sleep Lab cohort. Seizure frequency was determined for all epilepsy patients and depicted using a color gradient. **(A)** Brain age index (BAI) by group indicated a higher BAI in epilepsy, most notably in the generalizable group. The DREEM (n = 1,077) and Sleep Lab (n = 112) cohorts serve as controls with ^*^ < .05; ^**^p < .01;^***^p < .001. **(B)** Brain age and chronological age for all patients with epilepsy were compared, demonstrating a positive linear relationship with the intercept of 10.64 and a slope of 0.86, but no clear association based on the seizure frequency. **(C)** Brain age and chronological age for patients with generalizable epilepsy showed a positive linear relationship with an intercept of 9.47 and a slope of 0.90, but no clear association based on the seizure frequency. **(D)** Brain age and chronological age for patients with focal epilepsy showed a positive linear relationship with an intercept of 12.59 and slope of 0.78, but no clear association based on the seizure frequency.

The BAI was higher overall in patients with epilepsy (n = 138) compared to controls (Figure 2). The BAI [SE] was −0.05 [0.32] for all controls (Dreem and Sleep Lab), compared to 5.02 [0.43] in all epilepsy patients, 5.53 [0.48] for generalizable epilepsy patients (n = 106), and 3.34 [0.88] for focal epilepsy patients (n = 32). All patients with epilepsy had a statistically significant higher BAI compared to the Dreem (BAI -0.11 [0.34], *p* < 0.001) and Sleep Lab (BAI 0.53 [0.88], *p* < 0.001) control groups. Patients with generalizable seizures had a statistically significant higher BAI compared to the Dreem (*p* < 0.001) and Sleep Lab (*p* < 0.001) groups, as well as compared to the focal epilepsy group (*p* = 0.0304). Additionally, those with focal epilepsy had a higher BAI than the Dreem (*p* < 0.001) and Sleep Lab (*p* < 0.03) control groups (Figure 2A). The differences between the Dreem and Sleep Lab control groups were not significant (*p* = 0.56). Seizure frequency, which is a snapshot in time of how often seizures were occurring immediately prior to EMU admission, did not have an effect on the association between BA and chronological age in all patients with epilepsy (Figure 2B), generalizable epilepsy (Figure 2C), or focal epilepsy (Figure 2D). Additionally, no statistically significant difference was found between different focal seizure types (Supplementary Figure S3).

**FIGURE 3:**
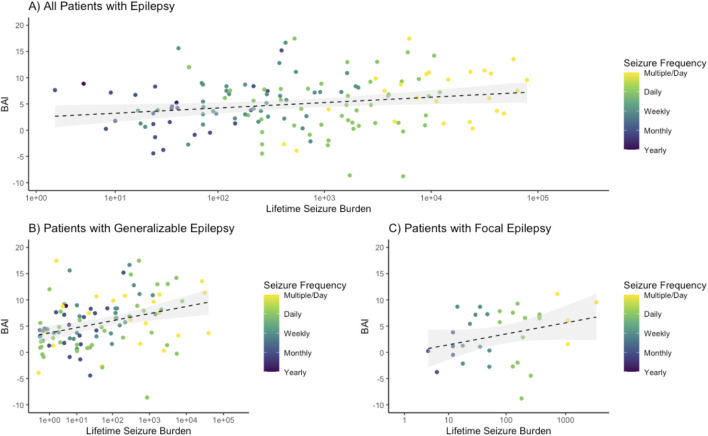
Brain age index is positively associated with lifetime seizure burden. The logarithm of the lifetime seizure burden was calculated based on the seizure frequency (per year) multiplied by the years of epilepsy and was then compared to the brain age index (BAI), demonstrating a weakly positive association. **(A)** For all epilepsy patients, a regression showed an R-squared of 0.0416 with an intercept of 2.16 and a slope of 0.45 with an associated *p*-value of 0.016. **(B)** For generalizable epilepsy patients, a regression showed an R-squared of 0.120 with an intercept of 3.30 and a slope of 0.59 with an associated *p*-value <0.001. **(C)** For non-generalizable (focal) seizure patients, a regression showed an R-squared of 0.0844 with an intercept of −0.76 and a slope of 0.92 with an associated *p*-value of 0.099.

Higher BAI (which is calculated as Brain Age Index = Brain Age–Chronological Age) was weakly associated with an increased lifetime seizure burden overall (*R* ^2^ = 0.0416, *p* = 0.02 (Figure 3A). The association was stronger in the generalizable epilepsy group, with an *R* ^2^ of 0.11 (*p* < 0.001) (Figure 3B), and weaker for focal epilepsy patients (*R* ^2^ = 0.09, *p* = 0.10 (Figure 3C). The average age and number of years with epilepsy were not significantly different between focal and generalizable seizure patients (see Table 1), suggesting that the associations above are not due to age alone. Supplementary Figure S4 demonstrates weaker but positive associations with other metrics of epilepsy severity, including years with epilepsy and seizure frequency immediately prior to EMU admission.

### Association between lifetime seizure burden and cognitive impairment

**FIGURE 4:**
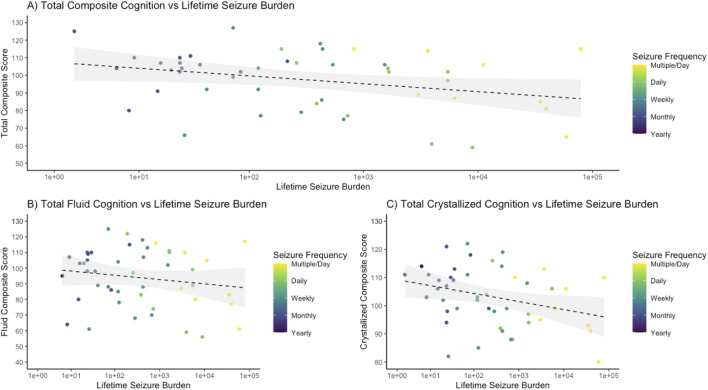
Cognitive functioning decreases with worsening lifetime seizure burden. The logarithm of the lifetime seizure burden was calculated based on the seizure frequency (per year) multiplied by the years of epilepsy and was then compared to scores on the NIH Toolbox cognitive function battery, demonstrating a negative association. **(A)** For total composite cognition, a regression demonstrated an R-squared of 0.130 with an intercept of 109.93 and a slope of −2.21 with an associated *p*-value of 0.011. **(B)** For total fluid cognition, a regression demonstrated an R-squared of 0.104 with an intercept of 107.70 and a slope of −2.30 with a *p*-value of 0.024. **(C)** For total crystallized cognition, a regression demonstrated an R-squared of 0.125 with an intercept of 110.58 and a slope of −1.46 with a *p*-value of 0.012.

There was a negative association between cognitive functioning and lifetime seizure burden. Of the 39-subject epilepsy cohort who underwent prospective cognitive testing, patients with an increased lifetime seizure burden had lower scores on the NIH Toolbox Cognitive Function Battery (Figure 4). Total composite cognition had the strongest negative association (*R* ^2^ = 0.130, *p* = 0.011), followed by total crystallized cognition (*R* ^2^ = 0.125, *p* = 0.012), and finally by total fluid cognition (*R* ^2^ = 0.104, *p* = 0.024). In comparison to healthy controls, there was a significant decrease in cognitive performance between epilepsy patients and controls, with mean (± standard deviation) values for total composite, fluid composite, and crystallized composite for epilepsy patients of 99.6 ± 16.6, 97.7 ± 18.9, and 102.6 ± 11.8 and for Sleep Lab controls of 109.2 ± 12.5, 105.8 ± 16.0, and 110.7 ± 9.6, with *p*-values of <0.001, 0.026, and <0.001 respectively.

### Association between BAI and cognitive impairment in epilepsy

**FIGURE 5:**
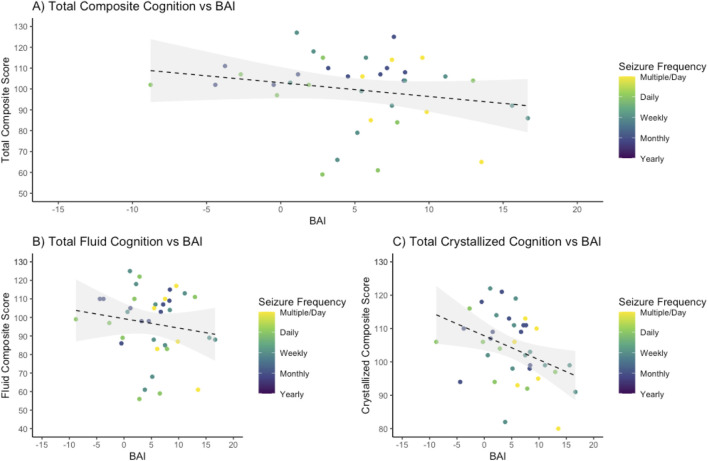
Higher crystalized cognitive functioning is negatively associated with brain age index. Brain age index (BAI) for all epilepsy patients was compared to scores on the NIH Toolbox cognitive function battery, demonstrating a negative association. **(A)** For total composite cognition, a regression demonstrated an R-squared of 0.0467 with an intercept of 103.00 and a slope of −0.66 with an associated *p*-value of 0.19. **(B)** For fluid cognition, a regression demonstrated an R-squared of 0.0146 with an intercept of 99.86 and a slope of −0.42 with an associated *p*­ value of 0.46. **(C)** For crystallized cognition, a regression demonstrated an R-squared of 0.102 with an intercept of 106.26 and a slope of −0.69 with a *p*-value of 0.048.

In the Sleep Lab control cohort, the BAI was not significantly correlated with total composite cognition (*p* = 0.75) or fluid cognition (*p* = 0.15) and had a significant negative correlation with crystallized cognition (*R* ^2^ = 0.0632, slope −0.1683, *p* = 0.002). In the 39-subject epilepsy group (patients who underwent prospective cognitive testing), higher BAI was associated with lower total crystallized cognition (*R* ^2^ = 0.102, *p* = 0.048), but not with total composite cognition (*R* ^2^ = 0.0467, *p* = 0.19) or fluid cognition (*R* ^2^ = 0.0146, *p* = 0.46) (Figure 5).

When comparing the association of BAI and cognition between patients with epilepsy (39-subject cohort with cognitive testing) and controls, the explained variance was higher in epilepsy patients for composite, fluid, and crystallized cognition (0.0467, 0.0146, and 0.102 in epilepsy patients vs. 0.0007, 0.0142, and 0.0632 in controls). The slope, or degree by which cognition worsens as BA increases, was steeper in epilepsy patients compared to healthy controls but did not meet statistical significance. ANCOVA between the slopes of the linear regressions for total composite, fluid, and crystallized cognition in epilepsy patients compared to healthy controls (−0.66, −0.42, and −0.69 compared to −0.0244, 0.1322, and −0.1683) indicated *p*-values of 0.12 for total composite, 0.28 for fluid composite, and 0.09 for crystallized composite cognition.

## Discussion

Our main findings are as follows: 1) conventional sleep architecture differs between patients with epilepsy and controls, with increased N2 and decreased N3 across all epilepsy cases, and an increase in REM in the generalizable and total groups; 2) BAI is substantially elevated in patients with epilepsy, with an average excess BA of 5.02 years; 3) BAI is higher in epilepsy patients with generalizable seizures compared to those with focal seizures that do not generalize; 4) BAI is positively associated with an increased lifetime seizure burden; 5) there is a significant negative association between lifetime seizure burden and fluid, crystallized, and composite cognition, and epilepsy patients had worse cognitive outcome measures than healthy controls, and 6) in epilepsy patients, higher BAI is associated with reduced crystallized cognition.

These findings are largely consistent with those of existing research on sleep changes in epilepsy. In our study, we found that the underlying sleep architecture in epilepsy differed from that of controls. We find that epilepsy patients have a greater proportion of N2 sleep and a lower proportion of N3 sleep, most notably in patients with focal seizures. REM sleep constituted a larger proportion of sleep in epilepsy patients overall, and particularly within the generalizable seizure subgroup, while the focal seizure group had a similar proportion of REM sleep compared to controls. Our finding that patients with epilepsy had increased REM sleep relative to controls was unexpected and could reflect changes in drug therapy (Tork et al., 2020; Yeh et al., 2021; Placidi et al., 2000; Bazil, 2003). It is important to note that our sample is not entirely representative of epilepsy patients as a whole, specifically that patients who undergo EMU stays tend to be medication-refractory (require more medications at higher doses) and during EMU stays often have variable titrations of medications to induce seizures and undergo multiple days of interrupted sleep during close clinical monitoring; this may affect the sleep architecture of this epilepsy patient cohort. Our cohort was otherwise representative of those included in studies identifying decreased REM in those with epilepsy, so this discrepancy warrants further investigation.

BAI was higher in epilepsy patients than in controls. The control group had a BAI near 0 (−0.1 and 0.53), while epilepsy patients had higher brain age indices, ranging from 3.3 in the focal seizure group to 5.53 in the generalizable seizure group. Of note, we did not expect the BAI in the control groups (Dreem and Sleep Lab to be exactly 0) since there could be other diseases or health issues that individuals might face that were not part of the exclusion criteria; the fact that the Sleep Lab control group, which contains patients who were referred to the hospital sleep lab for evaluation, demonstrated a slightly higher BAI that the Dreem control group, which consists of community-dwelling participants who are relatively healthy, supports this premise. Based on our understanding of generalizable epilepsy as disrupting large-scale brain networks, thus enabling seizure spread, it is reasonable to conclude that generalizable epilepsy is associated with more significant sleep changes due to widespread brain connectivity alterations (Focke et al., 2014; Peng et al., 2014). This could potentially explain the higher BAI of the generalizable seizure group compared to that of the healthy controls. Similarly, although there is a weak positive association, the generalizable seizure group demonstrated the strongest association between lifetime seizure burden and higher BAI, suggesting that ongoing, frequent generalizable seizures disrupt sleep networks, “aging” the sleeping brain. Epilepsy, both generalized and focal, is associated with sleep disruption and is known to disrupt the thalamic-generated sleep spindles, which are associated with memory consolidation (Myatchin and Lagae, 2007; Kramer et al., 2021; Schiller et al., 2022; Nobili et al., 2022). These seizure-based changes to the thalamus, and thalamo-cortical networks, might be more extensive in generalized and generalizable seizures and thus could provide a possible explanation for some of our findings, including why seizure generalizability, rather than seizure burden, appears to be relatively more associated with brain aging. Additionally, the relatively stronger association between lower crystallized cognition, which represents acquired knowledge (that requires memory consolidation), and higher BAI in both controls and patients with epilepsy provides further support for sleep spindle disruption as an etiology.

Compared to healthy controls, there was an 8- to 10-point statistically significant decrease across composite cognition measures for epilepsy patients. Increasing seizure burden was noted to be associated with poorer performance on cognitive testing. Prior studies have indicated that cognitive measures, like IQ, demonstrate a significantly negative association with the number of lifetime seizures, with a close to 20-point IQ drop between those with 2–10 lifetime seizures and those with >100 lifetime seizures (Aldenkamp and Bodde, 2005). The NIH Toolbox has been used in epilepsy to identify specify cognitive dysfunction, such as slower processing speed and multi-domain dysfunction in both focal and generalized epilepsies (Hwang et al., 2019; Garcia-Ramos et al., 2021). The general decrease across composite, crystallized, and fluid cognition seen in our study is consistent with the existing cognitive dysfunction literature in epilepsy and is worse in those with a higher seizure burden (Nickels et al., 2016; Hermann et al., 2021). Higher BAI scores were associated with poorer performance on cognitive testing, for composite, fluid, and crystallized cognition. Both controls and epilepsy patients demonstrated a similar negative association between increasing BAI and poorer cognition (with the notable exception of fluid cognition in healthy controls); epilepsy patients appeared to have a slightly stronger association that did not reach statistical significance. It is important to note also that while the controls were healthier and tended to have BAIs closer to 0, individual variability in health and other factors likely contributed to the negative association in the control group between increasing BAI and poorer crystallized cognition. Several subanalyses also indicated no significant effect on the BAI–cognition association based on seizure frequency, seizure burden, or seizure type. Overall, this study demonstrates that changes in sleep EEG in epilepsy patients that resemble the changes that occur with aging are associated with reduced cognitive performance.

Our study has several limitations. Since sleep EEG is generated by the brain itself, it is not possible to disentangle an underlying aging of the brain from a specific aging of sleep dynamics through this biomarker; they are inextricably linked. However, BAI is robust, in that it is primarily determined from EEG patterns during NREM sleep (especially N2 and N3), which would reflect brain physiology and not mental activity. An assessment of seizure frequency and associated seizure burden was conducted via a retrospective chart review, which may introduce errors based on patient recollection and physician recording; the seizure frequency used here is likely to be lower than that experienced by patients. Additionally, the sleep EEG recordings used to calculate the BAI were taken from patients who were in the EMU, which may introduce biases in sampling a population with more severe epilepsy than the general epilepsy population, often requiring more medications at higher doses, which can affect the BAI due to sleep disruption. During EMU hospitalization, seizure capture is the goal, so medications are weaned, and frequent seizures are often seen. Additionally, sleep is often interrupted in the hospital due to close clinical monitoring. The sleep EEG clips taken for the BAI calculation were not compared to medication levels or to a recently increased frequency of seizures. Nevertheless, prior studies of sleep and epilepsy have primarily investigated those with refractory epilepsy, which makes this sample representative of those found in the literature.

Additionally, while epilepsy cases were assessed during EMU hospitalization, control patients completed sleep assessments either in a sleep lab or at home, resulting in different environmental influences between the two cohorts. Furthermore, lab-based PSG and home-based EEG can use different numbers of electrodes, therefore reducing the quality of the assessment. However, our recent study suggests that even the use of two frontal electrodes (i.e., a limited number and a proxy for home-based EEG) has sufficient internal accuracy to assess BA (Sun et al., 2019). BAI, as an automated detection tool, can be biased by noise and systemic error; however, a trained EEG reader manually confirmed that we successfully excluded seizures. The trajectory of BAI over the course of life and any parallelism with change in cognition or even brain structure cannot be established from our data.

Subsequent investigations will seek to address these limitations through prospective analyses and a broader epilepsy cohort including those with less severe disease. Additionally, we will evaluate patients with epilepsy at onset for early prediction of future cognitive dysfunction on EEG. Future clinical implementation of the BAI could help with early detection of cognitive dysfunction in epilepsy and might assist in early interventions, like cognitive prehabilitation (Baxendale, 2020).

## Conclusion

Our study demonstrates that BAI, calculated from the sleep EEG, is associated with generalizable epilepsy and increased seizure burden. Cognitive dysfunction was shown to worsen with increased lifetime seizure burden, and, in epilepsy patients, a higher BAI was associated with poorer crystalized cognitive performance. Future work will investigate the association between sleep changes and BAI with other cognitive measures, change over time, post-surgical outcomes, and structural network abnormalities in epilepsy.

## Data Availability

The data analyzed in this study are subject to the following licenses/restrictions: The de-identified data to reproduce the results will be available after the time of publication. Data will be found at the Brain Data Science Platform (https://bdsp.io/). Requests to access these datasets should be directed to https://bdsp.io/.

## References

[B1] Aldenkamp A. P. Bodde N. (2005). Behaviour, cognition and epilepsy. Acta Neurol. Scand. Suppl. 182 (182), 19–25. 10.1111/J.1600-0404.2005.00523.X 16359429

[B2] Baxendale S. (2020). Cognitive rehabilitation and prehabilitation in people with epilepsy. Epilepsy and Behav. 106, 107027. 10.1016/J.YEBEH.2020.107027 32208338

[B3] Bazil C. W. (2003). Epilepsy and sleep disturbance. Epilepsy Behav. 4 (Suppl. 2), S39–S45. 10.1016/J.YEBEH.2003.07.005 14527482

[B4] Beghi E. (2016). Addressing the burden of epilepsy: many unmet needs. Pharmacol. Res. 107, 79–84. 10.1016/J.PHRS.2016.03.003 26952026

[B5] Beghi E. Giussani G. Nichols E. Abd-Allah F. Abdela J. Abdelalim A. (2019). Global, regional, and national burden of epilepsy, 1990–2016: a systematic analysis for the Global Burden of Disease Study 2016. Lancet Neurol. 18 (4), 357–375. 10.1016/S1474-4422(18)30454-X 30773428 PMC6416168

[B6] Cole J. H. Marioni R. E. Harris S. E. Deary I. J. (2019). Brain age and other bodily ‘ages’: implications for neuropsychiatry. Mol. Psychiatry 24 (2), 266–281. 10.1038/S41380-018-0098-1 29892055 PMC6344374

[B7] Cole J. H. Poudel R. P. K. Tsagkrasoulis D. Caan M. W. A. Steves C. Spector T. D. (2017). Predicting brain age with deep learning from raw imaging data results in a reliable and heritable biomarker. Neuroimage. 163, 115–124. 10.1016/J.NEUROIMAGE.2017.07.059 28765056

[B8] Cummings J. Apostolova L. Rabinovici G. D. Atri A. Aisen P. Greenberg S. (2023). Lecanemab: appropriate use recommendations. J. Prev. Alzheimer’s Dis. 10 (3), 362–377. 10.14283/jpad.2023.30 37357276 PMC10313141

[B9] Degiorgio C. M. Curtis A. Carapetian A. Hovsepian D. Krishnadasan A. Markovic D. (2020). Why are epilepsy mortality rates rising in the United States? A population-based multiple cause-of-death study. BMJ Open 10 (8), e035767. 10.1136/BMJOPEN-2019-035767 PMC7449302 32839157

[B10] Eggert T. Dorn H. Danker-Hopfe H. (2021). Nocturnal brain activity differs with age and sex: comparisons of sleep EEG power spectra between young and elderly men, and between 60–80-year-old men and women. Nat. Sci. Sleep. 13, 1611–1630. 10.2147/NSS.S327221 34584476 PMC8464589

[B11] Farina E. Raglio A. Giovagnoli A. R. (2015). Cognitive rehabilitation in epilepsy: an evidence-based review. Epilepsy Res. 109 (1), 210–218. 10.1016/J.EPLEPSYRES.2014.10.017 25524861

[B12] Focke N. K. Diederich C. Helms G. Nitsche M. A. Lerche H. Paulus W. (2014). Idiopathic-generalized epilepsy shows profound white matter diffusion—tensor imaging alterations. Hum. Brain Mapp. 35 (7), 3332–3342. 10.1002/HBM.22405 25050427 PMC6869818

[B13] Fountain N. B. Kim J. S. Lee S. I. (1998). Sleep deprivation activates epileptiform discharges independent of the activating effects of sleep. J. Clin. Neurophysiol. 15 (1), 69–75. 10.1097/00004691-199801000-00009 9502515

[B14] Garcia-Ramos C. Struck A. F. Cook C. Prabhakaran V. Nair V. Maganti R. (2021). Network topology of the cognitive phenotypes of temporal lobe epilepsy. Cortex 141, 55–65. 10.1016/J.CORTEX.2021.03.031 34029858 PMC8319135

[B15] Gibbon F. M. Maccormac E. Gringras P. (2019). Sleep and epilepsy: unfortunate bedfellows. Arch. Dis. Child. 104 (2), 189–192. 10.1136/ARCHDISCHILD-2017-313421 30266875 PMC6362435

[B16] Halasz P. Szucs A. (2017). Sleep, epilepsies, and cognitive impairment. Academic Press.

[B17] Hermann B. P. Struck A. F. Busch R. M. Reyes A. Kaestner E. McDonald C. R. (2021). Neurobehavioural comorbidities of epilepsy: towards a network-based precision taxonomy. Nat. Rev. Neurol. 17 (12), 731–746. 10.1038/s41582-021-00555-z 34552218 PMC8900353

[B18] Hogan J. Sun H. Paixao L. Westmeijer M. Sikka P. Jin J. (2021). Night-to-night variability of sleep electroencephalography-based brain age measurements. Clin. Neurophysiol. 132 (1), 1–12. 10.1016/J.CLINPH.2020.09.029 33248430 PMC7855943

[B19] Hu Y. Shan Y. Du Q. Ding Y. Shen C. Wang S. (2021). Gender and socioeconomic disparities in global burden of epilepsy: an analysis of time trends from 1990 to 2017. Front. Neurol. 12. 10.3389/FNEUR.2021.643450 PMC8085398 33935942

[B20] Hwang G. Dabbs K. Conant L. Nair V. A. Mathis J. Almane D. N. (2019). Cognitive slowing and its underlying neurobiology in temporal lobe epilepsy. Cortex 117, 41–52. 10.1016/J.CORTEX.2019.02.022 30927560 PMC6650302

[B21] Jaoude M. A. Sun H. Pellerin K. R. Pavlova M. Sarkis R. A. Cash S. S. (2020). Expert-level automated sleep staging of long-term scalp electroencephalography recordings using deep learning. Sleep 43 (11), zsaa112. 10.1093/SLEEP/ZSAA112 32478820 PMC7686563

[B22] Jing J. Ge W. Hong S. Fernandes M. Lin Z. Y. C. Yang C. (2023). Development of expert-level classification of seizures and rhythmic and periodic patterns during EEG interpretation. Neurology 100, e1750–e1762. 10.1212/WNL.0000000000207127 36878708 PMC10136013

[B23] Jing J. Sun H. Kim J. A. Herlopian A. Karakis I. Ng M. (2020). Development of expert-level automated detection of epileptiform discharges during electroencephalogram interpretation. JAMA Neurol. 77 (1), 103–108. 10.1001/JAMANEUROL.2019.3485 31633740 PMC6806668

[B24] Johnson E. L. Krauss G. L. Kucharska-Newton A. Albert M. S. Brandt J. Walker K. A. (2020). Dementia in late-onset epilepsy: the atherosclerosis risk in communities study. Neurology 95 (24), e3248. 10.1212/WNL.0000000000011080 33097597 PMC7836657

[B25] Kanner A. M. (2003). Depression in epilepsy: prevalence, clinical semiology, pathogenic mechanisms, and treatment. Biol. Psychiatry 54 (3), 388–398. 10.1016/S0006-3223(03)00469-4 12893113

[B26] Kanner A. M. (2016). Management of psychiatric and neurological comorbidities in epilepsy. Nat. Rev. Neurol. 12 (2), 106–116. 10.1038/NRNEUROL.2015.243 26782334

[B27] Keezer M. R. Sisodiya S. M. Sander J. W. (2016). Comorbidities of epilepsy: current concepts and future perspectives. Lancet Neurol. 15 (1), 106–115. 10.1016/S1474-4422(15)00225-2 26549780

[B28] Kramer M. A. Stoyell S. M. Chinappen D. Ostrowski L. M. Spencer E. R. Morgan A. K. (2021). Focal sleep spindle deficits reveal focal thalamocortical dysfunction and predict cognitive deficits in sleep activated developmental epilepsy. J. Neurosci. 41 (8), 1816–1829. 10.1523/JNEUROSCI.2009-20.2020 33468567 PMC8115887

[B29] Leone M. J. Sun H. Boutros C. L. Liu L. Ye E. Sullivan L. (2021). HIV increases sleep-based brain age despite antiretroviral therapy. Sleep 44 (8), zsab058–9. 10.1093/SLEEP/ZSAB058 33783511 PMC8361332

[B30] Louis E. K. St (2011). Sleep and epilepsy: strange bedfellows no more. Minerva Pneumol. 50 (3), 159–176. Available at: https://mayoclinic.pure.elsevier.com/en/publications/sleep-and-epilepsy-strange-bedfellows-no-more (Accessed March 27, 2023). 23539488 PMC3608109

[B31] Malow B. A. Selwa L. M. Ross D. Aldrich M. S. (1999). Lateralizing value of interictal spikes on overnight sleep-EEG studies in temporal lobe epilepsy. Epilepsia 40 (11), 1587–1592. 10.1111/J.1528-1157.1999.TB02044.X 10565587

[B32] Murray C. J. L. Vos T. Lozano R. Naghavi M. Flaxman A. D. Michaud C. (2012). Disability-adjusted life years (DALYs) for 291 diseases and injuries in 21 regions, 1990-2010: a systematic analysis for the Global Burden of Disease Study 2010. Lancet 380 (9859), 2197–2223. 10.1016/S0140-6736(12)61689-4 23245608

[B33] Myatchin I. Lagae L. (2007). Sleep spindle abnormalities in children with generalized spike-wave discharges. Pediatr. Neurol. 36 (2), 106–111. 10.1016/J.PEDIATRNEUROL.2006.09.014 17275662

[B34] Nascimento F. A. Nath A. R. (2020). Teaching NeuroImages: epileptiform K-complexes in genetic generalized epilepsy: common but underappreciated. Neurology 94 (19), E2072–E2073. 10.1212/WNL.0000000000009414 32317351

[B35] National Institutes of Health NIH toolbox for assessment of neurological and behavioral function | blueprint. Available at: https://neuroscienceblueprint.nih.gov/resources-tools/blueprint-resources-tools-library/nih-toolbox-assessment-neurological-and (Accessed January 9, 2023).

[B36] Nickels K. C. Zaccariello M. J. Hamiwka L. D. Wirrell E. C. (2016). Cognitive and neurodevelopmental comorbidities in paediatric epilepsy. Nat. Rev. Neurol. 12 (8), 465–476. 10.1038/nrneurol.2016.98 27448186

[B37] Nobili L. Frauscher B. Eriksson S. Gibbs S. A. Halasz P. Lambert I. (2022). Sleep and epilepsy: a snapshot of knowledge and future research lines. J. Sleep. Res. 31 (4), e13622. 10.1111/JSR.13622 35487880 PMC9540671

[B38] Paixao L. Sikka P. Sun H. Jain A. Hogan J. Thomas R. (2020). Excess brain age in the sleep electroencephalogram predicts reduced life expectancy. Neurobiol. Aging 88, 150–155. 10.1016/J.NEUROBIOLAGING.2019.12.015 31932049 PMC7085452

[B39] Peng S. J. Harnod T. Tsai J. Z. Huang C. C. Ker M. D. Chiou J. C. (2014). Through diffusion tensor magnetic resonance imaging to evaluate the original properties of neural pathways of patients with partial seizures and secondary generalization by individual anatomic reference atlas. Biomed. Res. Int. 2014, 419376. Published online. 10.1155/2014/419376 24883310 PMC4026917

[B40] Petit D. Gagnon J. F. Fantini M. L. Ferini-Strambi L. Montplaisir J. (2004). Sleep and quantitative EEG in neurodegenerative disorders. J. Psychosom. Res. 56 (5), 487–496. 10.1016/j.jpsychores.2004.02.001 15172204

[B41] Placidi F. Diomedi M. Scalise A. Marciani M. G. Romigi A. Gigli G. L. (2000). Effect of anticonvulsants on nocturnal sleep in epilepsy. Neurology 54 (5 Suppl. 1), S25–S32. Available at: http://intl.neurology.org/cgi/content/full/54/5_suppl_1/S25 (Accessed January 15, 2023). 10718681

[B42] Rauh R. Schulze-Bonhage A. Metternich B. (2022). Assessment of anxiety in patients with epilepsy: a literature review. Front. Neurol. 13, 836321. 10.3389/FNEUR.2022.836321 35547374 PMC9081800

[B43] Rossi K. C. Joe J. Makhija M. Goldenholz D. M. (2020). Insufficient sleep, electroencephalogram activation, and seizure risk: Re-evaluating the evidence. Ann. Neurol. 87 (6), 798–806. 10.1002/ANA.25710 32118310

[B44] Schiller K. Avigdor T. Abdallah C. Sziklas V. Crane J. Stefani A. (2022). Focal epilepsy disrupts spindle structure and function. Sci. Rep. 12, 11137. 10.1038/s41598-022-15147-0 35778434 PMC9249850

[B45] Sen A. Jette N. Husain M. Sander J. W. (2020). Epilepsy in older people. Lancet 395 (10225), 735–748. 10.1016/S0140-6736(19)33064-8 32113502

[B46] Seneviratne U. Cook M. D’Souza W. (2016). Epileptiform K-complexes and sleep spindles: an underreported phenomenon in genetic generalized epilepsy. J. Clin. Neurophysiol. 33 (2), 156–161. 10.1097/WNP.0000000000000239 26587665

[B47] Stuart E. A. (2010). Matching methods for causal inference: a review and a look forward. Stat. Sci. 25 (1), 1–21. 10.1214/09-STS313 20871802 PMC2943670

[B48] Sun H. Paixao L. Oliva J. T. Goparaju B. Carvalho D. Z. van Leeuwen K. G. (2019). Brain age from the electroencephalogram of sleep. Neurobiol. Aging 74, 112–120. 10.1016/J.NEUROBIOLAGING.2018.10.016 30448611 PMC6478501

[B49] Tork M. A. Rashed H. R. Elnabil L. Salah-Eldin N. Elkhayat N. Abdelhady A. A. (2020). Sleep pattern in epilepsy patients: a polysomnographic study. Egypt. J. Neurology, Psychiatry Neurosurg. 56 (1), 5. 10.1186/s41983-019-0141-4

[B50] Weintraub S. Dikmen S. S. Heaton R. K. Tulsky D. S. Zelazo P. D. Slotkin J. (2014). The cognition battery of the NIH toolbox for assessment of neurological and behavioral function: validation in an adult sample. J. Int. Neuropsychol. Soc. 20 (6), 567–578. 10.1017/S1355617714000320 24959840 PMC4103959

[B51] Ye E. Sun H. Leone M. J. Paixao L. Thomas R. J. Lam A. D. (2020). Association of sleep electroencephalography-based brain age index with dementia. JAMA Netw. Open 3 (9), e2017357. 10.1001/JAMANETWORKOPEN.2020.17357 32986106 PMC7522697

[B52] Ye E. M. Sun H. Krishnamurthy P. V. Adra N. Ganglberger W. Thomas R. J. (2023). Dementia detection from brain activity during sleep. Sleep 46 (3), zsac286–11. 10.1093/SLEEP/ZSAC286 36448766 PMC9995788

[B53] Yeh W. C. Lai C. L. Wu M. N. Lin H. C. Lee K. W. Li Y. S. (2021). Rapid eye movement sleep disturbance in patients with refractory epilepsy: a polysomnographic study. Sleep. Med. 81, 101–108. 10.1016/J.SLEEP.2021.02.007 33647761

