# Supplementary material for: Epilepsy is associated with the accelerated aging of brain activity in sleep
Source: Front Physiol. 2024 Nov 28;15:1458592. doi: 10.3389/fphys.2024.1458592 (PMC11634596; doi:10.3389/fphys.2024.1458592)
Supplement: Supplementary file 1 [file DataSheet3.docx]

Supplemental

**Epilepsy is Associated with the Accelerated Aging of Brain Activity in Sleep**

Running Head: Epilepsy and Sleep-Based Brain Aging

Peter N. Hadar ^a*^, MD, MSc, Mike Westmeijer^b,c*^, MSc, Haoqi Sun^c*^, PhD,
Erik-Jan Meulenbrugge^c^, MSc, Jin Jing^c^, PhD, Luis Paixao^c^, BMBCh, MSc, Ryan A. Tesh^c^, BSc, Madalena Da Silva Cardoso^d^, MSc, Pierrick Arnal^e^, PhD, Rhoda Au^f^, PhD, Chol Shin^g,h^, MD, PhD, Soriul Kim^g^, PhD, Robert J. Thomas^i^, MD, Sydney S. Cash^a^, MD, PhD**, M. Brandon Westover^c^, MD, PhD**

*^a.^  Dept. of Neurology, Massachusetts General Hospital (MGH), Boston, MA, USA ^b.^* *Utrecht University, Utrecht, The Netherlands
^c.^ Dept. of Neurology, Beth Israel Deaconess Medical Center, Boston, MA*

*^d.^ Dept. of Radiology, NYU-Langone Medical Center, New York, NY
^e.^ Dreem, Paris France*

*^f.^ Dept. of Epidemiology, Boston University School of Medicine, Boston, MA*

*^g.^ Institute of Human Genomic Study, College of Medicine, Korea University, Seoul, Republic of Korea*

*^h.^ Biomedical Research Center, Korea University Ansan Hospital, Ansan, Republic of Korea
^i.^ Dept. of Medicine, Division of Pulmonary, Critical Care & Sleep, Beth Israel Deaconess
 Medical Center, Boston, MA*

* Co-first authors

** Co-senior authors

**
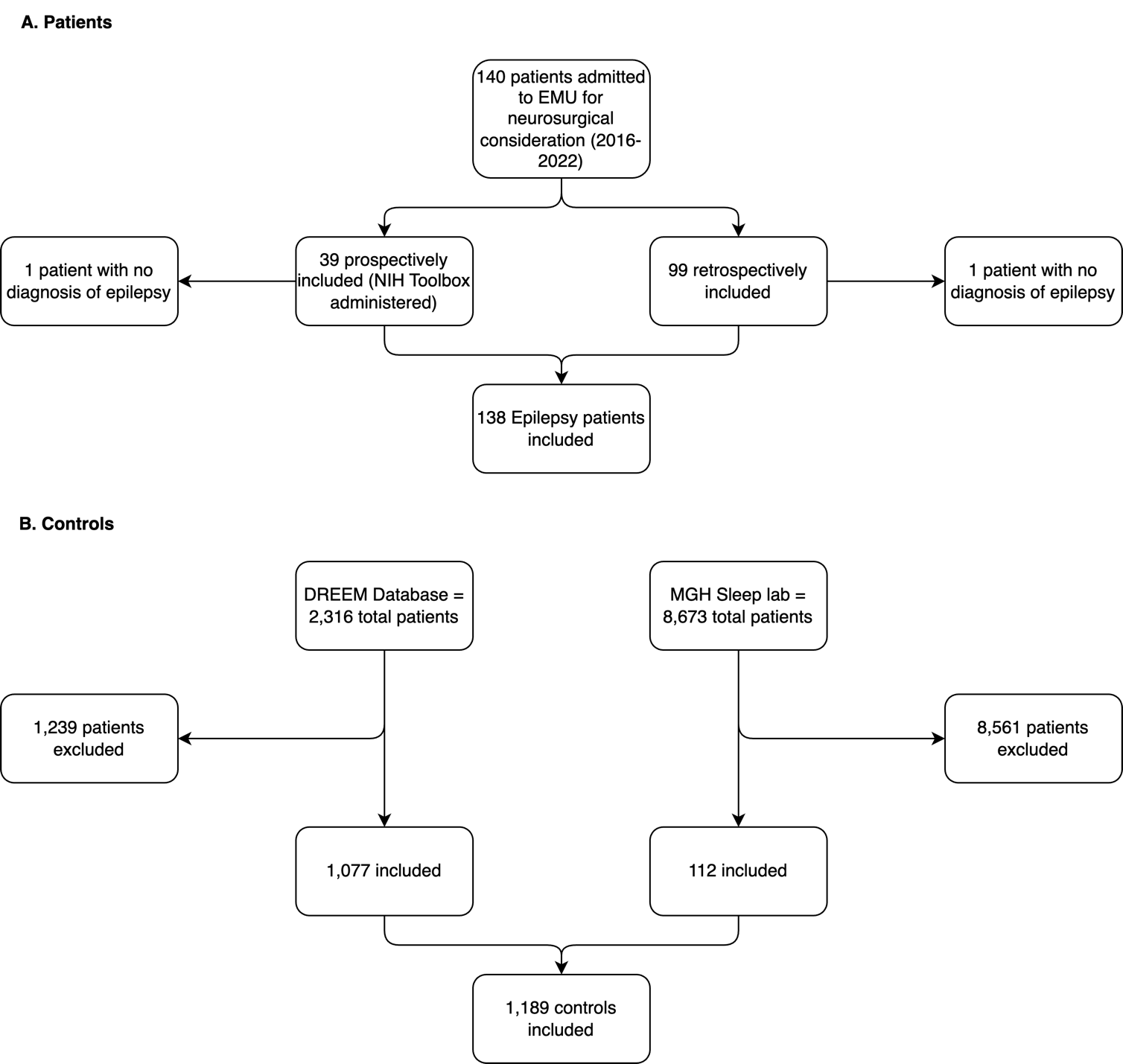
**

**Supplementary Fig. 1:** **Patient Selection Flowchart for Patients (A) and Controls (B).** Exclusion criteria for controls from DREEM study included shift worker, sleep apnea, restless leg syndrome, circadian rhythm disturbance, sleep walking, hypersomnia, narcolepsy, cataplexy, REM sleep behavior disorder, insomnia, depression, or other pathology. Exclusion criteria for controls from the MGH Sleep lab included no NIH Toolbox Cognitive Battery, body mass index > 30kg/m^2^, apnea-hypopnea index > 5/hour, periodic limb movement index > 10/hour, sleep latency > 30 minutes, wakefulness after sleep onset > 30 minutes, use of antidepressants/opiates/benzodiazepines, diagnosed brain conditions (i.e., stroke, meningioma, dementia, mild cognitive impairment), trisomy 21, and Huntington’s disease.


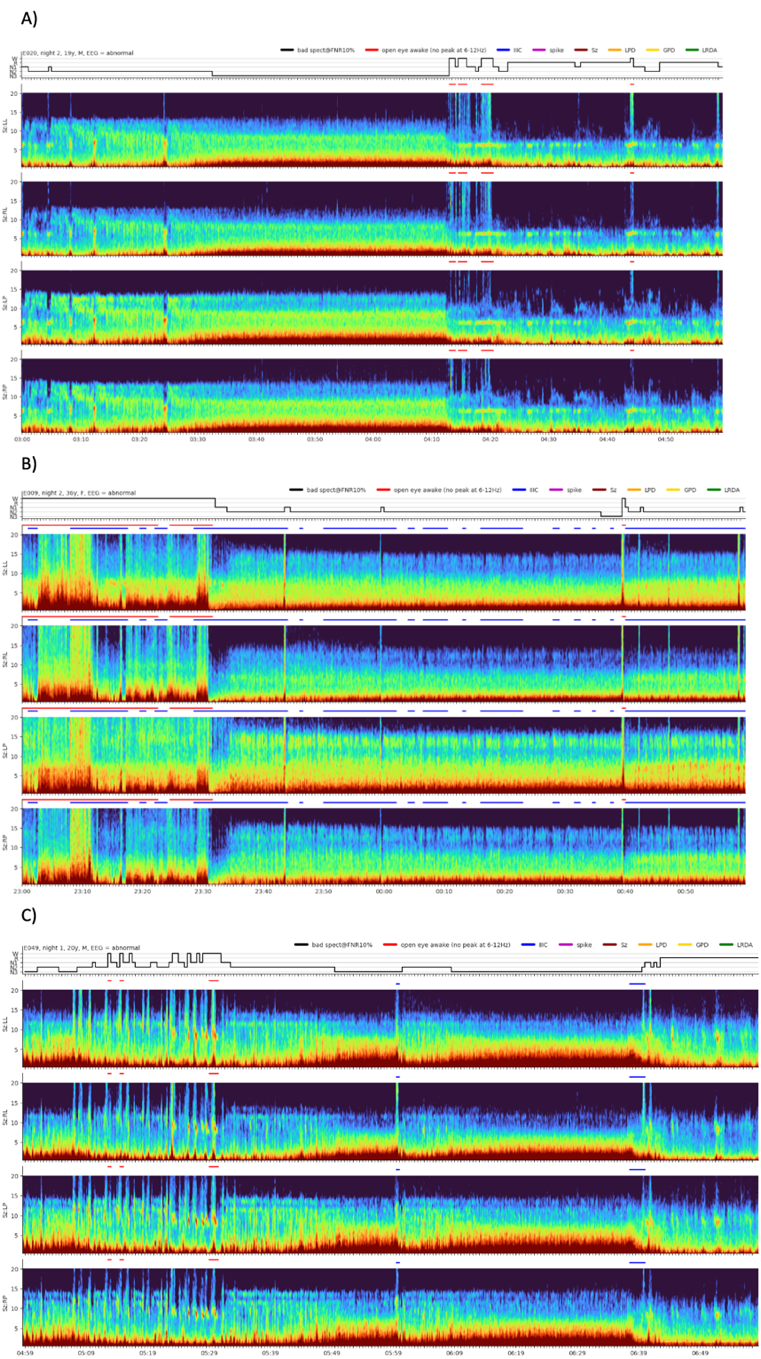


**Supplementary Fig. 2:** **Representative Hypnograms and Spectrograms.** A) Shows the hypnogram for a patient with epilepsy and BAI of 0.35 (near the average for controls). B) For a patient with epilepsy and BAI of 6.13 (near average for all epilepsy patients). C) For a patient with epilepsy and BAI of 16.7 (elevated).


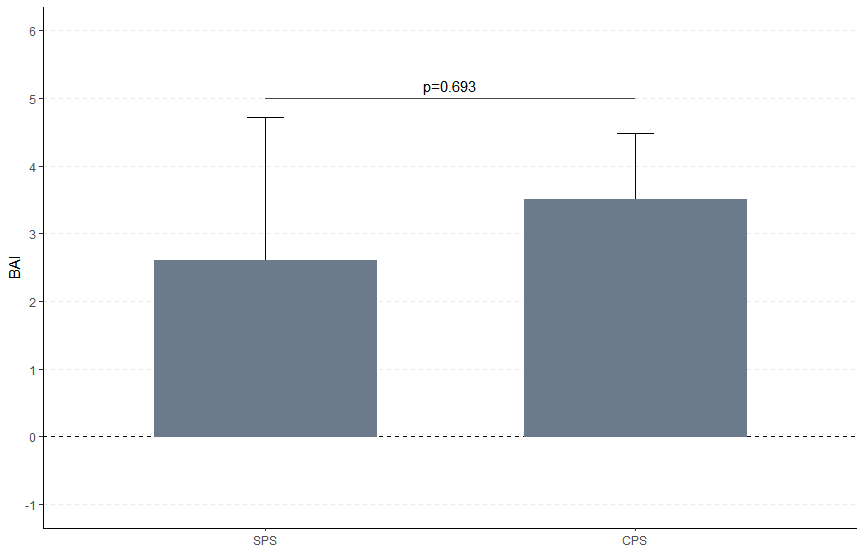


**Supplementary Fig. 3:** **No Statistically Significant Difference in Brain Age Index (BAI) between Focal Seizure Types.** The focal aware seizures (simple partial seizures (SPS) with n=6) had a mean BAI of 2.60 and a standard error of 2.12, while the focal impaired awareness seizure (complex partial seizures (CPS) n=26) had a mean BAI of 3.51 and a standard error of 0.98. Although there appeared to be a higher BAI in the CPS groups compared to the SPS group, this did not reach statistical significance.

**
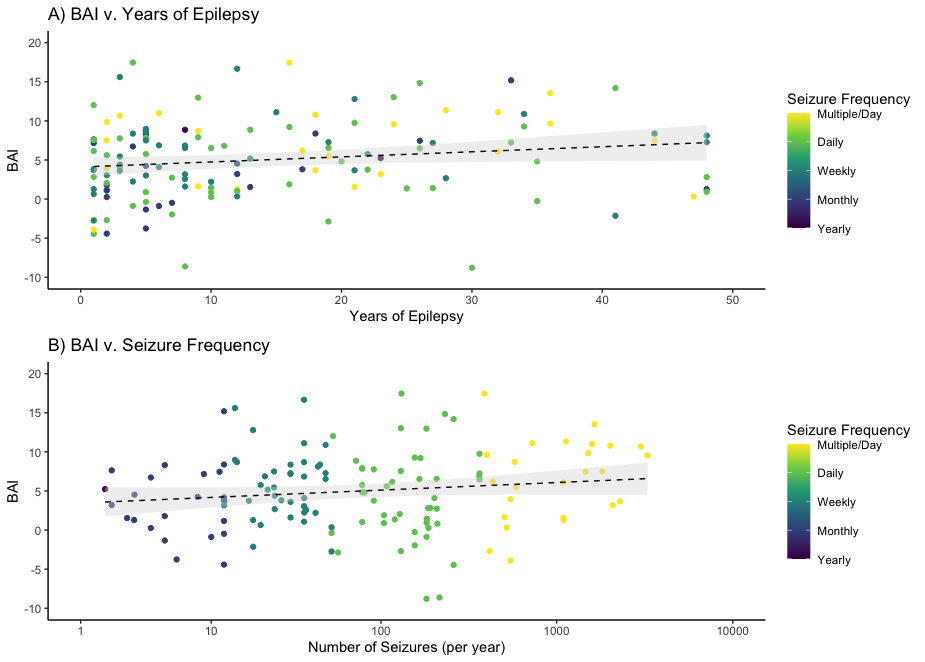
**

**Supplementary Fig. 4:** **BAI is Positively Associated with Years of Epilepsy.** A) The years that a patient has epilepsy is slightly positively associated with the Brain Age Index (BAI), regression showed an R-squared of 0.0297 with an intercept of 4.09 and a slope of 0.065 with an associated p-value of 0.043. B) For the logarithm of seizure frequency immediately prior to EMU admission, a regression showed an R-squared of 0.0159 with an intercept of 3.42 and a slope of 0.37 with an associated p-value of 0.14.
